# Prime-boost immunization by both DNA vaccine and oncolytic adenovirus expressing GM-CSF and shRNA of TGF-β2 induces anti-tumor immune activation

**DOI:** 10.18632/oncotarget.15008

**Published:** 2017-02-02

**Authors:** So Young Kim, Dongxu Kang, Hye Jin Choi, Yeonsoo Joo, Joo-Hang Kim, Jae J. Song

**Affiliations:** ^1^ Institute for Cancer Research, Yonsei University College of Medicine, Seoul, Korea; ^2^ Department of Oncology, Affiliated Hospital of Yanbian University, Yanji, Jilin Province, P.R. China; ^3^ Department of Internal Medicine, Yonsei University College of Medicine, Seoul, Korea; ^4^ Severance Biomedical Science Institute, Yonsei University College of Medicine, Seoul, Korea; ^5^ CHA Bundang Medical Center, CHA University, Seongnam, Korea

**Keywords:** oncolytic adenovirus, MART1, DNA vaccine, GM-CSF, TGF-β2

## Abstract

A successful DNA vaccine for the treatment of tumors should break established immune tolerance to tumor antigen. However, due to the relatively low immunogenicity of DNA vaccines, compared to other kinds of vaccines using live virus or protein, a recombinant viral vector was used to enhance humoral and cellular immunity. In the current study, we sought to develop a novel anti-cancer agent as a complex of DNA and oncolytic adenovirus for the treatment of malignant melanoma in the C57BL/6 mouse model. MART1, a human melanoma-specific tumor antigen, was used to induce an increased immune reaction, since a MART1-protective response is required to overcome immune tolerance to the melanoma antigen MelanA. Because GM-CSF is a potent inducer of anti-tumor immunity and TGF-β2 is involved in tumor survival and host immune suppression, mouse GM-CSF (mGM-CSF) and shRNA of mouse TGF-β2 (shmTGF-β2) genes were delivered together with MART1 via oncolytic adenovirus. MART1 plasmid was also used for antigen-priming. To compare the anti-tumor effect of oncolytic adenovirus expressing both mGM-CSF and shmTGF-β2 (Ad^GshT^) with that of oncolytic adenovirus expressing mGM-CSF only (Ad^G^), each virus was intratumorally injected into melanoma-bearing C57BL/6 mice. As a result, mice that received Ad^GshT^ showed delayed tumor growth than those that received Ad^G^. Heterologous prime-boost immunization was combined with oncolytic Ad^GshT^ and MART1 expression to result in further delayed tumor growth. This regression is likely due to the following 4 combinations: MART1-derived mouse melanoma antigen-specific immune reaction, immune stimulation by mGM-CSF/shmTGF-β2, tumor growth inhibition by shmTGF-β2, and tumor cell-specific lysis via an oncolytic adenovirus. Immune activation was mainly induced by mature tumor-infiltrating dendritic cell (TIDC) and lowered regulatory T cells in tumor-infiltrating lymphocytes (TIL). Taken together, these findings demonstrate that human MART1 induces a mouse melanoma antigen-specific immune reaction. In addition, the results also indicate that combination therapy of MART1 plasmid, together with an oncolytic adenovirus expressing MART1, mGM-CSF, and shmTGF-β2, is a promising candidate for the treatment of malignant melanoma.

## INTRODUCTION

The incidence of malignant melanoma is increasing worldwide [1], and is the fifth highest incidence among cancers in the US and the UK [2]. Although early primary melanomas can be cured surgically, this cancer can rapidly become fatal, following the development of metastasis. Therapeutic agents such as ipilimumab, which targets the cytotoxic T lymphocyte-associated protein 4 (CTLA4), and vemurafenib, which targets mutations that activate the B- Rapidly Accelerated Fibrosarcoma (RAF) gene, have previously been developed for malignant melanoma. However, while these agents take a short amount of time to respond to treatment, they lack high cure rates [3–6]. Therefore, the development of new treatments is inescapable, in order to improve the prognosis for malignant melanoma patients. In this study, granulocyte-macrophage colony-stimulating factor (GM-CSF), shRNA against transforming growth factor-β (TGF-β), and melanoma antigen recognized by T cells 1 (MART1) were administered to mice to determine the effectiveness of immunotherapy as a complex form of DNA vaccine and armed oncolytic adenovirus on melanoma.

Some of the most potent inducers of anti-tumor immunity include the following: GM-CSF, a molecule that enhances immune responses by inducing proliferation, maturation, and migration of dendritic cells (DCs); expansion and differentiation of B and T lymphocytes; and direct recruitment of natural killer (NK) cells. On the contrary, GM-CSF is also known to enhance the expansion of myeloid derived suppressor cells (MDSC) [7]. The systemic use of recombinant GM-CSF is compromised by side effects, as well as the induction of potentially harmful MDSCs [8–10]. Furthermore, the efficacy of systemic recombinant GM-CSF treatment is limited, due to the presence of only a low local concentration of GM-CSF in tumors [11]. Therefore, local GM-CSF production by cancer cells could ensure a sufficient local concentration, while minimizing systemic exposure. This means GM-CSF is an appealing molecule for local delivery to tumors, which would also be particularly useful in the context of oncolytic adenoviruses.

TGF-β signal plays important roles in tumor cell proliferation, differentiation, angiogenesis, collagen deposition, tumor invasion, and metastasis [12, 13]. In addition, TGF-β produced by cancer cells, or by stroma cells, stimulates MDSC to repress the immune response by certain cell types, including NK cells, DCs, macrophages, and T cells [7, 14]. The isoforms of TGF-β are TGF-β1, TGF-β2, and TGF-β3 [15]. While TGF-β1 is expressed in epithelial, endothelial, hematopoietic, and connective tissue cells, TGF-β2 is expressed in epithelial and neuronal cells, and TGF-β3 is expressed primarily in mesenchymal cells [16–18]. Specifically, TGF-β2 increases cytokine-associated immunosuppression [19], antagonizes NK cells, lymphokine-activated killer (LAK) cells, and GM-CSF-induced DC maturation [20–25], and contributes as a main activator of MDSC [26]. Thus, to stimulate the immune response efficiently with GM-CSF, TGF-β blockade is indispensable [23]. In addition, oncolytic viral infection of tumor cells induces the generation of anti-tumor immune responses via innate and adaptive anti-tumor immunity [27]. To boost anti-tumoral immunity, immunostimulatory cytokines, such as GM-CSF, have been loaded to oncolytic viruses [28–34]. In order to determine whether the anti-tumoral immunity of GM-CSF is enhanced by TGF-β inactivation by oncolytic viruses, we designed an oncolytic adenovirus expressing GM-CSF along with shRNA of TGF-β2 to examine the maximal anti-tumoral activity induced by optimal immune reaction. Blockade of TGF-β signaling also enhances tumor antigen-specific T cell activation [35], which provides a basis for the strong connection of TGF-β inhibition with immunization using tumor-specific antigen.

Plasmid DNA immunization has potential advantages, compared with traditional protein vaccination, as DNA induces strong cytotoxic T lymphocyte (CTL) and T helper 1 (Th1) responses, prolonged antigen expression, and resistance of the antigen source to antibody-mediated clearance [36–41]. To induce a mouse melanoma antigen-specific immune response, we utilized MART1, a human melanocyte lineage-specific protein, which is expressed by 75–100% of melanomas but not by other cell or tumor types [42]. It has been speculated that activation of multiple immune pathways by antigens, delivered using different vectors, synergistically enhances the immune response to target antigens [43]. One approach that has proven to be particularly effective in eliciting a robust immune response is priming with plasmid DNA and boosting with a replication-incompetent adenovirus vector that encodes the same antigen [44].

In this study, we demonstrated prime-boost immunization by both DNA vaccine and oncolytic adenovirus, expressing that MART1, GM-CSF, and shRNA of TGF-β2 acting cooperatively induces both tumor-specific immune activation and general immune activation with oncolysis to have a pronounced anti-tumor effect.

## RESULTS

### Establishment of a mouse cell line with higher rate of adenoviral infection and replication

**Figure 1:**
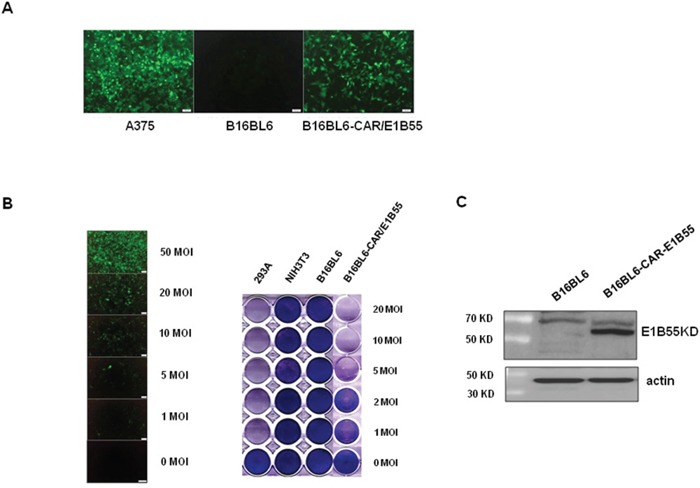
Infectivity of adenovirus in B16BL6-CAR/E1B55 cell line **A**. A375 (human melanoma cell line), B16BL6 (mouse melanoma cell line), and B16BL6-CAR/E1B55 were infected with adenovirus-GFP at an MOI of 50. After 48 h, GFP expression was detected. **B**. The B16BL6-CAR/E1B55 cell line was infected with adenovirus-GFP at various MOIs (Left). To compare the oncolytic activity induced by Ad3484-CMVp-ΔE1B, cancer and normal cells were infected with each virus at an MOI of 1 to 20. When 293A cells infected with one of the viruses at an MOI of 1 exhibited complete cell lysis, all the remaining cells on the plate were fixed with 4% paraformaldehyde and stained with 0.5% crystal violet (Right). **C**. E1B-55K protein was detected by using E1B-55K polyclonal antiserum from one of selected clone of B16BL6-CAR-E1B55K cell line.

Compared with human cancer cells, most mouse cancer cells have lower infection efficiency with adenovirus, likely because mouse cells do not express the coxsackievirus and adenovirus receptor (CAR) [45, 46]. In addition, the replication rate of adenovirus is very low, which limits the effectiveness of oncolytic adenovirus in killing mouse cancer cells [47]. To overcome these limitations, the B16BL6-CAR/E1B55 mouse melanoma cell line was developed [47]. This cell line expresses both the CAR and adenoviral E1B55 genes, and thus, exhibits enhanced infectivity by adenovirus (Figure 1A). The replication-dependent cytotoxic effects of adenovirus in B16BL6-CAR/E1B55 cells were quantitatively assessed using an *in vitro* cytopathic effect (CPE) assay. The replication of oncolytic adenovirus was induced in B16BL6-CAR/E1B55 cells in a multiplicity of infection (MOI)-dependent manner (Figure 1B, Left), and was clearly revealed in the *in vitro* cytopathic effect (CPE) assay (Figure 1B, Right). The expression of E1B-55KD protein in the structure of B16BL6-CAR/E1B55 was confirmed using newly produced E1B-55KD polyclonal antibody (Figure 1C).

### TGF-β downregulation in melanoma cell

**Figure 2:**
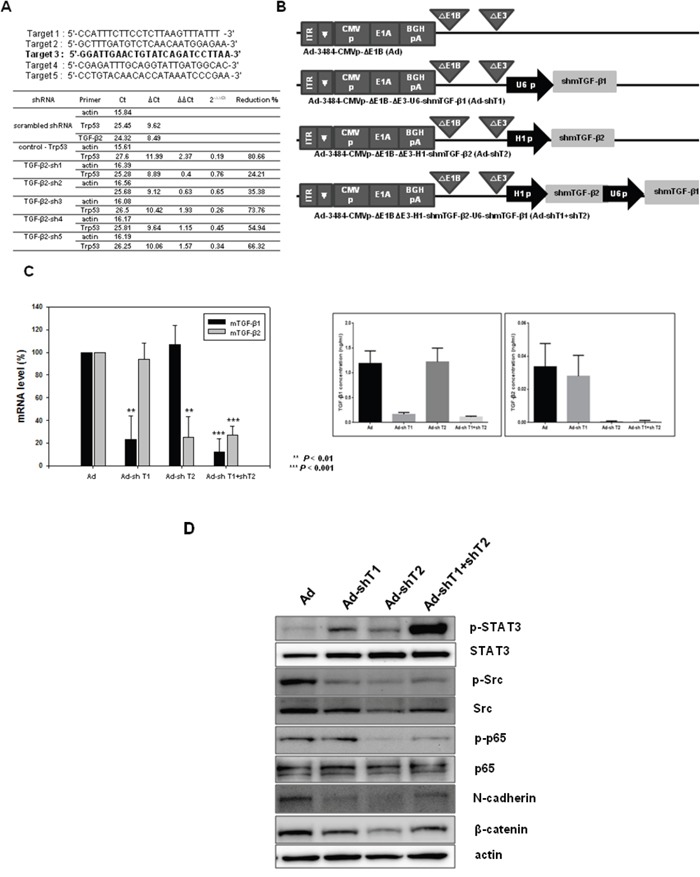
Screening of mouse TGF-β2 and changes in signaling molecules by adenovirus expressing shmTGF-β **A**. Screening of mouse TGF-β2 shRNAs. Sequences of shRNA oligomers targeting mouse TGF-β2 are shown with the selected target sequence indicated in bold (Top). The candidate oligomers for each target and the positive control shRNA were transfected into B16F10 cells. The knockdown efficiency of each oligomer was measured using quantitative real-time PCR to amplify mouse TGF-β2. Relative expression levels of mouse TGF-β2 were plotted after normalization to the scrambled shRNA as a negative control (Bottom). **B**. Ad-3484-CMVp-ΔE1B is a replication-competent adenovirus used as a control (Ad). This adenovirus contains the E1A gene controlled by the CMV promoter but lacks the E1B gene. Ad-3484-CMVp-ΔE1B-ΔE3-U6-shmTGF-β1 (Ad-shT1) and Ad-3484-CMVp-ΔE1B-ΔE3-H1-shmTGF-β2 (Ad-shT2) are composed of the shmTGF-β1 or shmTGF-β2 genes in the E3 region of Ad-3484-CMVp-ΔE1B, respectively. Ad-3484-CMVp-ΔE1B-ΔE3-H1-shmTGFβ2-U6-shmTGF-β1 (Ad-sh1+shT2) is composed of the shmTGF-β1 and shmTGF-β2 genes in the E3 region of Ad-3484-CMVp-ΔE1B. **C**. Relative expression levels of mTGF-β1 and mTGF-β2 mRNA. Oncolytic Ad (Ad), Ad-shTGFβ1 (Ad-shT1), Ad-shTGFβ2 (Ad-shT2), or Ad-shTGFβ1+shTGFβ2 (Ad-shT1+shT2) virus were injected into B16BL6-CAR/E1B55 cells at an MOI of 100. The knockdown efficiency of these viruses was measured by quantitative real-time PCR of mTGF-β1 or mTGF-β2 (Left). Protein levels of mTGF-β1 and mTGF-β2. Oncolytic Ad (Ad), Ad-shTGFβ1 (Ad-shT1), Ad-shTGFβ2 (Ad-shT2), or Ad-shTGFβ1+shTGFβ2 (Ad-shT1+shT2) virus were injected into B16BL6-CAR/E1B55 cells at an MOI of 100. The knockdown efficiency of these viruses was measured by ELISA of TGF-β1 (Right, top) or TGF-β2 (Right, bottom). **D**. B16BL6-CAR/E1B55 cells were injected with oncolytic Ad (Ad), Ad-shTGFβ1 (Ad-shT1), Ad-shTGFβ2 (Ad-shT2), or Ad-shTGFβ1+shTGFβ2 (AdshT1+shT2) virus at an MOI of 100. Two days later, the endogenous expression levels of various signaling molecules were detected by western blot.

Real-time PCR confirmed the downregulation of TGF-β transcripts, induced by adenovirus expressing shRNA against mouse TGF-β1, TGF-β2, or both TGF-β1 and TGF-β2 in B16BL6-CAR/E1B55 cells. Five oligomers of TGF-β2 shRNA, as well as control shRNA (shRNA against scrambled sequence), were also validated using real-time PCR after selection of appropriate target sequences; in addition, the target sequence with maximal repression was identified. The target of TGF-β1 has been described previously [48]. As shown in Figure 2A, among five validated TGF-β2 shRNAs (designated as TGF-β2 sh1–5), TGF-β2 sh3 elicited the greatest reduction of TGF-β2 mRNA levels (74%). To construct an oncolytic adenovirus, TGF-β shRNA sequences were inserted into the pSP72ΔE3-U6 (or H1) E3 shuttle vector to yield Ad-3484-CMVp-ΔE1B-U6-shmTGF-β1 (Ad-shT1), Ad-3484-CMVp-ΔE1B-H1-shmTGF-β2 (Ad-shT2), or Ad-3484-CMVp-ΔE1B-U6-shmTGF-β1-H1-shmTGF-β2 (Ad-shT1+shT2). Ad-shT1 construct specifically decreased TGF-β1 mRNA levels, while Ad-shT2 specifically decreased TGF-β2 mRNA levels (Figure 2C, Left). Furthermore, the actual protein level of TGFβ1 or TGFβ2 dowregulation by shRNA was also significantly decreased by the adenovirus that expressed shRNAs targeting TGF-β1 or TGF-β2, respectively (Figure 2C, Right). However, based on Figure 2D, downregulation of TGF-β isotype 2, other than isotype 1 or even both of isotypes 1 and 2, greatly reduced the cellular level of signaling molecules such as phospho-p65, phospho-Src, N-cadherin and β-catenin which are involved in cancer cell survival and metastasis.

### Development of oncolytic adenovirus expressing both mGM-CSF and shmTGF-β2

**Figure 3:**
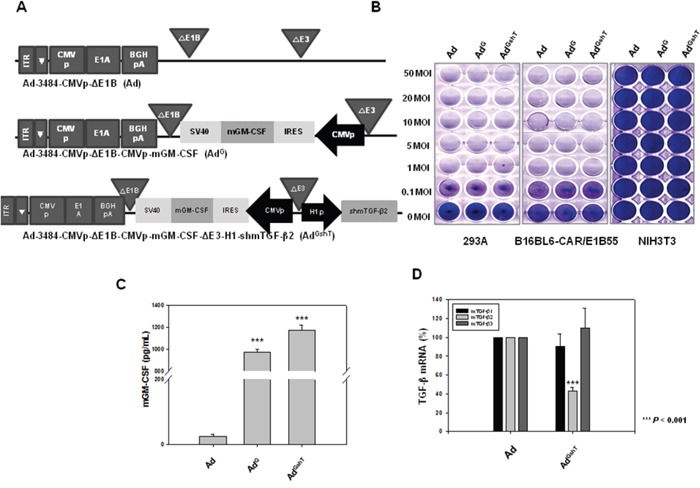
Recombinant adenoviruses expressing mGM-CSF and shmTGF-β2 **A**. Schematic representation of adenovirus vectors expressing mGM-CSF and shmTGF-β2. Ad-3484-CMVp-ΔE1B-CMVp-mGM-CSF (Ad^G^) is composed of the mGM-CSF gene in the E1 region, and Ad-3484-CMVp-ΔE1B-CMVp-mGM-CSF-ΔE3-H1-shmTGF-β2 (Ad^GshT^) is composed of the shmTGF-β2 gene in the E3 region of Ad-3484-CMVp-ΔE1B. **B**. Oncolytic activity of these viruses was analyzed using an *in vitro* CPE assay. Cancer and normal cells were infected with each virus at an MOI of 0.1 to 50. To examine the level of mGM-CSF and mTGF-β2 mRNA expression, B16BL6-CAR/E1B55 cells were infected with virus at a MOI of 50. Two days after injection, mGM-CSF expression levels were measured in the culture supernatants by ELISA **C**. and mTGF-β mRNA was estimated by RT-PCR **D**.

As shown in Figure 2D, downregulation of mTGF-β2, rather than mTGF-β1, clearly reduced the expression of survival/metastasis-promoting molecules. As a result, effective protection from tumor challenge was expected to be conferred by the combined treatment of mGM-CSF and shRNA against mTGF-β2. To develop the mGM-CSF-expressing recombinant oncolytic adenovirus, mGM-CSF gene was inserted into the E1 region of oncolytic adenovirus to yield Ad^G^. In addition, Ad^GshT^ was generated as an oncolytic adenovirus, expressing both mGM-CSF and shRNA against mTGF-β2 (Figure 3A). The oncolytic ability of these replication-competent adenoviruses was analyzed by an *in vitro* CPE assay of various cell lines (293A, B16BL6-CAR/E1B55, and NIH3T3 cells), after cells were infected with oncolytic Ad, Ad^G^, or Ad^GshT^ at different MOIs. As shown in Figure 3B, all replication-competent adenoviruses induced increasing CPE in melanoma cells (B16BL6-CAR/E1B55), but not in normal cells (NIH3T3).

### Oncolytic adenovirus expressing GM-CSF and shRNA of TGF-β delayed tumor growth concomitant with immune stimulation in a mouse model

**Figure 4:**
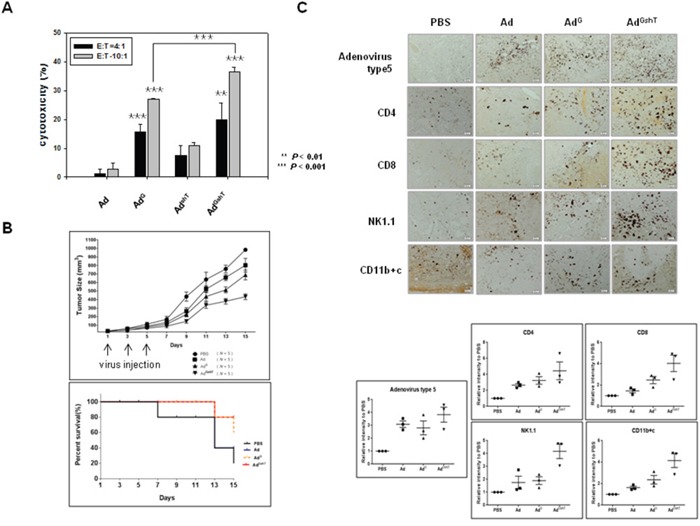
The anti-tumor effect of adenoviruses expressing mGM-CSF and shmTGF-β2 The anti-tumor effect of oncolytic Ad-GMCSF, Ad-shTGFβ2 or Ad-GMCSF-shTGFβ2 was confirmed by *ex vivo* (A) and *in vivo* (B) experiments. **A**. B16BL6-CAR/E1B55 cells infected with each virus at an MOI of 50 were incubated with splenocytes isolated from 3 mice of each group of C57BL/6 for 4 h. The splenocyte cytotoxic activity was measured by an LDH assay. E:T means the ratio of total number of effector cells (splenocytes) to target cells (infected B16BL6-CAR/E1B55 cells). **B**. C57BL/6 tumor-bearing mice were treated with intratumoral injections of 1 × 10^9^ PFU/50 μL of adenoviruses on days 1, 3, and 5. Tumor volume was monitored and recorded every 2 days until the end of the study. Values represent the mean ± SE (5 animals per group)(Top). Overall survival was determined throughout a 15-day time course (Bottom). **C**. Representative immunohistochemical analysis of recombinant adenovirus infected tumor sections. 3 mice of each group of C57BL/6 tumor-bearing mice were treated with intratumoral injections of 1 × 10^9^ PFU/50 μL of adenoviruses on days 1, 3, and 5. Tumors were collected at day 11 for histological analysis. Paraffin- sections of tumor tissue were stained with anti-adenovirus type 5, anti-CD4, anti-CD8, anti-NK1.1 antibodies, and anti-CD11b+c. The dark purple spots indicate.adenovirus type 5, CD4, CD8, NK/NKT and DC cells, respectively. NK1.1 is a key marker of NK and NKT cells. CD11b is a key marker of macrophages and CD11c is a key marker of dendritic cells (Top). Results are given as the relative intensity of adenovirus type 5, CD4, CD8, NK/NKT and DC cells to PBS of each 3 mouse. Horizontal black bars indicate mean values (Bottom).

To demonstrate the expression levels of both mGM-CSF and mTGF-β2, B16BL6-CAR/E1B55 cells were infected with Ad^GshT^ at an MOI of 50. Two days after infection, the cellular mRNA levels of mGM-CSF and mTGF-β2 were examined. A significant increase in the secreted amount of mGM-CSF (Figure 3C) and a significant reduction of mTGF-β2 mRNA levels (Figure 3D) were observed. To confirm that an enhanced anti-cancer immune response was induced by recombinant adenovirus expressing both mGM-CSF and shRNA against mTGF-β2, *ex vivo* and *in vivo* tests were performed. The cytotoxic activity of splenocytes against mouse melanoma cells, infected with various oncolytic adenovirus (Ad^G^, Ad^shT^, Ad^GshT^), was measured with an lactate dehydrogenase (LDH) assay. The oncolytic Ad-GMCSF-shTGFβ2-infected cells induced enhanced splenocyte anti-tumor activity, compared to cells infected with oncolytic Ad-GMCSF or Ad-shTGFβ2 only (Figure 4A). To determine whether the same effects were induced in an animal model of melanoma, C57BL/6 mice were subcutaneously injected with B16BL6-CAR/E1B55 melanoma cells. When tumors reached a range of 50–60 mm^3^, mice were intratumorally injected with PBS, oncolytic Ad as a control (Ad), oncolytic Ad-GMCSF (Ad^G^), or oncolytic Ad-GMCSF-shTGFβ2 (Ad^GshT^) on days 1, 3, and 5. As shown in Figure 4B, control tumors that received PBS exhibited the fastest growth, and tumors infected with both oncolytic Ad and oncolytic Ad-GMCSF were even grown rapidly, while oncolytic Ad-GMCSF-shTGFβ2 showed an effective delayed tumor growth. This enhanced anti-tumor effect was attributed to both the stimulation of a non-specific immune reaction by mGM-CSF expression and the downregulation of mTGF-β2. However, meaningful tumor regression was not observed even in the oncolytic Ad-GMCSF-shTGFβ2-infected mice group.

To identify the types of infiltrating immune cells in tumor tissues, tumor sections were examined by immunohistochemistry using anti-CD4, anti-CD8, anti-NK1.1, or CD11b+c antibody for the detection of CD4^+^ T cell, CD8^+^ T cell, NK cell or NK T cell, and macrophage or dendritic cell, respectively (Figure 4C). As a result, infiltration of CD8^+^ T cells, CD4^+^ T cells, NK cells, and NK T cells in tumor tissues increased, after infection of oncolytic Ad-GMCSF-shTGFβ2, compared to the infection of oncolytic control adenovirus, but not significantly.

### Induction of mouse melanoma antigen-specific immune activity by human MART1 plasmid

**Figure 5:**
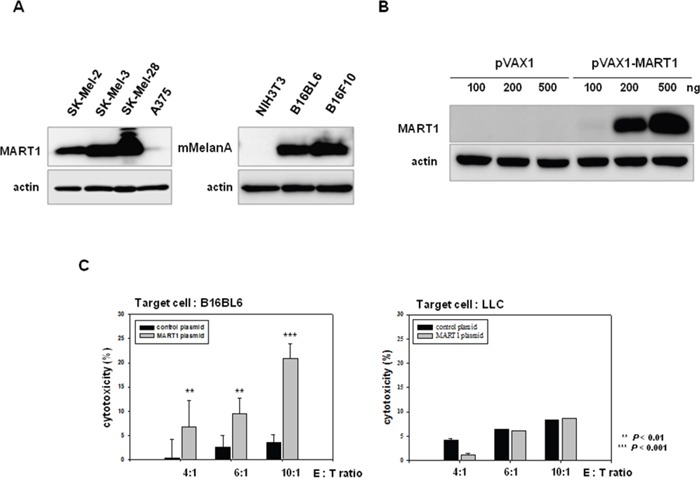
Effects of the human MART1 plasmid on mouse melanoma antigen-specific immune priming **A**. MART1 and mMelanA expression was detected in various human and mouse melanoma cells (except NIH3T3 as a negative control) by western blot. **B**. MART1 translational levels were assessed in B16BL6 cells after transfection with pVAX1 or pVAX-MART1. **C**. Splenocytes were co-cultured with B16BL6 or LLC cells for 4 h after collection from 4 mice of each group injected with control plasmid or MART1 plasmid. The cytotoxicity of splenocytes was then determined with an LDH assay.

Ribas *et al*. (2000) previously showed that DCs, that were genetically modified to express human MART1, generated murine MelanA-specific anti-tumor immune responses in a B16 melanoma model [49]. These findings imply that stimulation of MART1 will induce a MelanA-specific immune response in our mouse melanoma model. We examined the endogenous level of MART1 or MelanA in various melanoma cell lines. MART1 was expressed in human melanoma cell lines (SK-MEL-2, SK-MEL-3, and SK-MEL-28), whereas murine MelanA, which has 68.8% homology with MART1 [49], was expressed in mouse melanoma cell lines (B16BL6 and B16F10), but not in mouse normal cells (NIH3T3; Figure 5A). Accordingly, we decided to use MART1 as a target of immune priming/boosting for immunotherapy of melanoma. The expression of MART1 protein increased in MART1 plasmid-transfected mouse melanoma cells, in a dose-dependent manner (Figure 5B). To induce immune activation, 50 μg (per 25 g of mouse) of MART1 plasmid in a total volume of 50 μl saline was injected intramuscularly into the rear quadriceps of C57BL/6 mice, since muscle cells injected with plasmid DNA produce large amounts of gene-encoded protein [36, 50]. Injections of MART1 plasmid were given three times at one-week intervals. One week after the final injection, mice were sacrificed, and their spleens were removed. To determine the effect of immune priming by MART1, splenocytes were isolated, and spontaneous LDH release was measured after co-culture of the splenocytes with B16BL6 mouse melanoma cells. Cytotoxic activity of the splenocytes isolated from mice injected with MART1 plasmid was effectively enhanced, compared with that of the splenocytes isolated from mice injected with control plasmid (Figure 5C). Moreover, the enhancement of splenocyte cytotoxic activity was dependent on the ratio between effector cells (splenocytes) and target cells (B16BL6 cells) (Figure 5C, Left). The immune activation effect, which was induced by MART1 plasmid treatment, was not observed in the Lewis lung carcinoma (LLC) cell line (Figure 5C, Right), which is a mouse lung cancer cell line that does not express MelanA. This result indicates that injection of human MART1 plasmid primes mouse MelanA-specific immunity, as it induces the development of an immune cell population that is cross-reactive to both human MART-1 and mouse MelanA.

### Development of adenovirus expressing MART1, GM-CSF, and shTGF-β2

**Figure 6:**
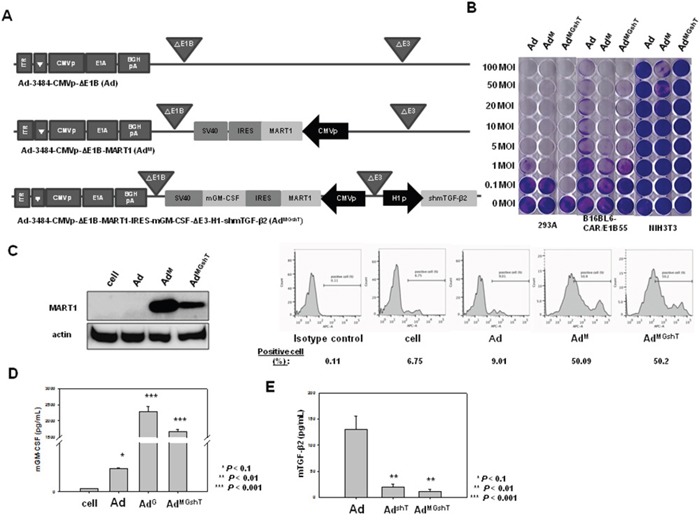
Recombinant adenovirus vectors expressing MART1, mGM-CSF, and shmTGF-β2 **A**. Ad3484-CMVp-ΔE1B-CMVp-MART1 (Ad^M^) is composed of the MART1 gene in the E1 region of Ad3484-CMVp-ΔE1B. Ad3484-CMVp-ΔE1B-MART1-IRES-mGM-CSF-ΔE3-H1-shmTGF-β2 (Ad^MGshT^) is composed of the MART1 and mGM-CSF genes in the E1 region and shmTGF-β2 gene in the E3 region of Ad3484-CMVp-ΔE1B. **B**. The oncolytic activity of these viruses was analyzed by an *in vitro* CPE assay. Cancer and normal cells were infected with each virus at an MOI of 0.1 to 100. **C**. Left). B16BL6-CAR/E1B55 cells were infected with Ad, Ad^M^, Ad^MGshT^ at an MOI of 50. Two days later, the endogenous expression level of MART1 was detected by western blot. MART1 cell-surface expression levels were also detected by flow cytometric analysis (C, Right). To examine mGM-CSF levels, B16BL6-CAR/E1B55 cells were infected with Ad, Ad^G^, Ad^MGshT^ at a MOI of 50. Two days after injection, mGM-CSF expression levels were measured in the culture supernatants by ELISA **D**. To examine mTGF-β2 protein levels, B16BL6-CAR/E1B55 cells were infected with Ad, Ad^shT^, Ad^MGshT^ at a MOI of 50. Two days after injection, mTGF-β2 protein levels were measured in the culture supernatants by ELISA **E**.

In an attempt to produce stronger and longer-lasting immune responses than those created by Ad^GshT^, recombinant pVAX1-MART1 plasmid, and recombinant oncolytic adenovirus expressing MART1, mGM-CSF, and shRNA of mTGF-β2 were administered. Ad-3484-CMVp-ΔE1B-MART1 (Ad^M^) was constructed with the purpose of inducing mouse melanoma antigen-specific immune priming and boosting. Furthermore, Ad3484-CMVp-ΔE1B-MART1-IRES-mGM-CSF-ΔE3-H1-shmTGF-β2 (Ad^MGshT^) was constructed in order to simultaneously enhance the mouse melanoma antigen-specific immune response, non-specific general immune response, and suppression of cancer cell growth. MART1 and mGM-CSF, which were part of an internal ribosome entry site (IRES) expression cassette, were inserted into the E1 region of adenovirus genome, while shmTGF-β2 was inserted into the E3 region (Figure 6A). The oncolytic activity of the recombinant adenoviruses was verified by an *in vitro* CPE assay (Figure 6B). Before progressing to animal tests to further investigate the anti-tumor effects of the Ad^MGshT^, expressions of oncolytic adenovirus-arming MART1, mGM-CSF and shmTGF-β2 genes were examined. B16BL6-CAR/E1B55 cells were infected with oncolytic control virus (Ad), oncolytic virus expressing MART1 (Ad^M^), or oncolytic virus expressing MART1, GM-CSF, and shRNA of TGF-β2 (Ad^MGshT^) at an MOI of 50. Two days after infection, MART1 protein was detected by western blotting, and the results showed a significant increase in the endogenous cellular levels of the virally transduced MART1 protein (Figure 6C, Left). Furthermore, MART1 protein, which is located on the surface of melanoma cells, was detected by flow cytometric analysis, and the surface expression of MART1 increased on MART1 gene-containing virus-infected cells (Figure 6C, Right). To quantify the expression level of mGM-CSF induced by infection with recombinant adenovirus, B16BL6-CAR/E1B55 cells were infected with Ad^c^, Ad^G^, or Ad^MGshT^ virus at an MOI of 50. An enzyme-linked immunosorbent assay (ELISA) was then performed to estimate the mGM-CSF protein concentration of cell supernatants. The mGMCSF gene-containing virus-infected cells exhibited significantly increased secretion of the virally transduced mGM-CSF protein (Figure 6D), while the level of secreted mTGF-β2 protein significantly decreased in the shRNA of TGF-β2 gene-containing virus-infected cells (Figure 6E).

### Anti-tumor effect of MART1 plasmid with oncolytic adenovirus expressing GM-CSF and shRNA of TGF-β2

**Figure 7:**
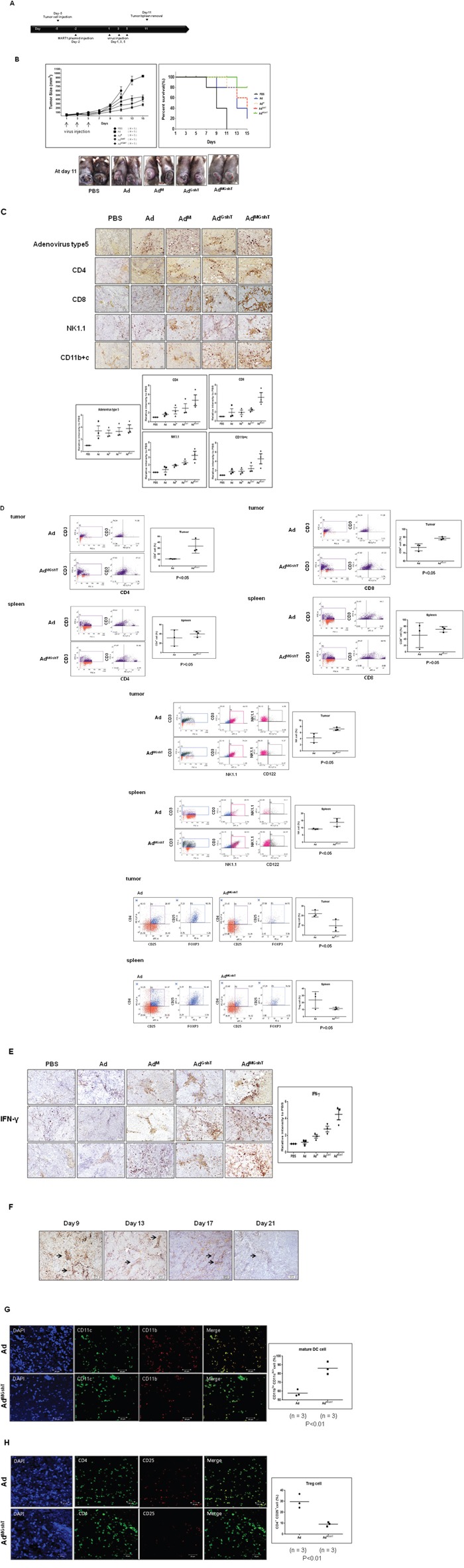
Anti-tumor effect of the combination treatment of MART1 plasmid with Ad, Ad^M^, Ad^GshT^, and Ad^MGshT^ **A**. Diagram of the experimental design. C57BL/6 mice were injected with 7 × 10^5^ B16BL6-CAR/E1B55 cells in 100 μL on day -5 and injected intramuscularly with 50 μg/50 μL of MART1 plasmid into the rear quadriceps on day -2. C57BL/6 tumor-bearing mice were treated with intratumoral injections of 1 × 10^9^ PFU/50 μL of adenovirus on day 1, 3, and 5. **B**. Tumor volume was monitored and recorded every 2 days until the end of the study. Values represent the mean ± SE (5 animals per group) (Top, Left). Overall survival was determined throughout a 15-day time course (Top, Right). Photographs of C57BL/6 tumor-bearing mice treated with virus were obtained at day 11 after virus infection (Bottom). **C**. Representative immunohistochemical analysis of recombinant adenovirus-infected tumor sections was done as follows. 3 animals per group of C57BL/6 mice were injected with 7 × 10^5^ B16BL6-CAR/E1B55 cells/100 μL on day -5 and then treated with intramuscular injections of 50 μg/50 μL of MART1 plasmid into the rear quadriceps on day -2. C57BL/6 tumor-bearing mice were treated with intratumoral injections of 1 × 10^9^ PFU/50 μL of various kinds of adenovirus on day 1, 3, and 5. Tumors were collected at day 11 for histological analysis. Paraffin sections of tumor tissue were stained with anti-adenovirus type 5, anti-CD8, anti-CD4, anti-NK1.1, anti-CD11b+c (Top) Results are given as the relative intensity of adenovirus type 5, CD4, CD8, NK/NKT and DC cells to PBS of each 3 mouse. Horizontal black bars indicate mean values (Bottom). **D**. Flow cytometric analysis of various types of tumorigenic or splenic immune cells after combination treatment of MART1 plasmid with Ad or Ad^MGshT^. 3 animals per group of mice bearing B16BL6-CAR/E1B55 cells were sacrificed and tumorigenic or splenic immune cells were isolated after combination treatment of MART1 plasmid with Ad or Ad^MGshT^ as indicated in detail in (A). To set the gate, T-cell gating of positive CD3 using Jurkat cell was performed. Each cell was then costained with anti-CD3 and anti-CD8 (or anti-CD4) for the detection of T cells. Otherwise, each cell was then costained with anti-NK1.1 and anti-CD122 after gating with anti-CD3 and anti-NK1.1 for the detection of NKT cells or for the detection of regulatory T cells, each cell was then costained with anti-CD25 and anti-FOXP3 after gating with anti-CD4 and anti-CD25. Numbers in inset are percentage of cells in a given quadrant. All flow cytometric results are representative of three experiments (Left, Middle). Right results are the average percentage of corresponding immune cells in each 3 mouse. Horizontal black bars indicate mean values. **E**. Immunohistochemical analysis was performed as indicated in (C) by staining with anti-IFN-γ antibody (Left). Results are given as the relative intensity of IFN-γ to PBS of each 3 mouse. Horizontal black bars indicate mean values (Right). **F**. IHC using Ad5 antibody was performed to examine the residual adenovirus at the indicated day after infection of oncolytic control adenovirus was administered intratumorally (1 × 10^9^ PFU per tumor in 50 μl of PBS) on days 1, 3, and 5. Representative confocal immunofluorescence staining of tumor sections was done as described in Materials and methods to confirm the increased mature TIDCs (**G**., green color) or decreased regulatory T cells (**H**., orange color) after combination treatment of MART1 plasmid with Ad^MGshT^ (Left). TIDC or regulatory T cells were counted on images taken from multiple fields per mouse (n = 3/group). Results are given as the average percentage of CD11b^lo^CD11c^hi+^ cells or CD4^+^CD25^+^ cells in each 3 mouse. Horizontal black bars indicate mean values (Right).

We expected that treatment with MART1 plasmid together with the oncolytic adenovirus expressing MART1, mGM-CSF, and shmTGF-β2 (Ad^MGshT^) would generate a stronger anti-tumor effect against B16BL6-CAR/E1B55 *in vivo*, as this method was designed to induce priming-boosting and stimulation of immunity and oncolysis. Figure 7A shows experimental conditions for the heterologous prime-boost immunization, designed to induce an anti-tumor effect using MART1 DNA and Ad^MGshT^ virus. As shown in Figure 7B, the growth rate of tumors in mice immunized with MART1 plasmid and Ad was slightly delayed than that in mice immunized with MART1 plasmid and PBS. In contrast, tumors of mice receiving both MART1 plasmid and Ad^MGshT^ had a significantly delayed growth rate compared to those of mice treated with either Ad, Ad^M^ or even Ad^GshT^, although tumor regression was not induced (Figure 7B).

To analyze the infection rate of adenovirus vector and the infiltration of immune cells to tumor tissues following mouse injection with MART1 plasmid and adenovirus, we performed a histologic analysis of the tumor site. As shown in Figure 7C, many of the tumor tissues in mice injected with MART1 plasmid + Ad, MART1 plasmid + Ad^M^, or MART1 plasmid + Ad^MGshT^ displayed expression of adenovirus-specific protein, whereas mice in the PBS-treated group did not. Furthermore, in the mice that were immunized with MART1 plasmid + Ad^MGshT^, many tumor tissues exhibited increased lymphocytic infiltration compared with that of the mice immunized with MART1 plasmid + Ad or MART1 plasmid + Ad^M^. To identify the types of immune cells that had infiltrated into the tumor tissues, tumor sections were examined by immunohistochemistry using anti-CD4, anti-CD8, anti-NK1.1 or anti-CD11b+c monoclonal antibodies. Slightly higher frequencies of CD8^+^ T cells, NK cells, NKT cells, dendritic cells and macrophages were observed in the tumors treated with MART1 plasmid + Ad^MGshT^ when compared to those of mice treated with MART1 plasmid + Ad or MART1 plasmid + Ad^M^ (Figure 7C).

### Enhanced infiltration of tumor tissues by both of T cells and dendritic cells after MART1 plasmid + Ad^MGshT^

To investigate further immune activation by MART1 plasmid + Ad^MGshT^, tumor-infiltrating or splenic immune cells were examined. As shown in Figure 7D, tumor-infiltrating CD4^+^, CD8^+^ T and NKT cells increased after MART1 plasmid + Ad^MGshT^, instead, tumor-infiltrating CD4^+^CD25^+^FoxP3^+^ regulatory T cells were decreased. However, elevated or decreased levels of corresponding immune cells were not significant in spleen. Then, we examined IFN-γ expression at the tumor site whether the tumor-infiltrating immune cells had higher immune activities depending on the combinations. Consequently, the highest concentration of IFN-γ was clearly observed in tumor tissue after treatment with MART1 plasmid + Ad^MGshT^ (Figure 7E). However, adenoviral production gradually decreased until after three weeks (Figure 7F). To more clearly distinguish the phenotype of tumor-infiltrating dendritic cells (TIDCs), TIDCs were regarded as either mature dendritic cells by higher intensity CD11c^+^/lower intensity CD11b^+^, or tolerogenic dendritic cells by lower intensity CD11c^+^/higher intensity CD11b^+^. As shown in Figure 7G, virtually all of the CD11c^+^ TIDCs also co-expressed CD11b^+^ on their surfaces, as previously indicated by Berhanu *et al*. [51], and most dendritic cells were maturated after treatment with MART1 plasmid + Ad^MGshT^. Similar result was also obtained in CD4^+^ T cells, with decreasing amount of regulatory T cells after treatment with MART1 plasmid + Ad^MGshT^ (Figure 7H).

**Figure 8:**
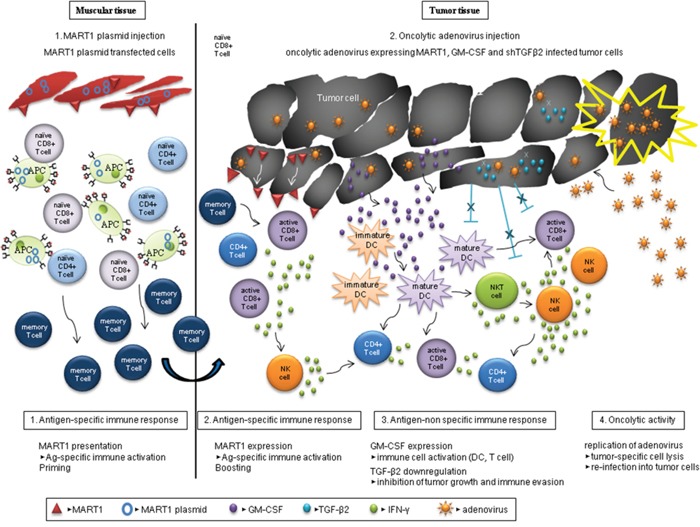
Schematic heterologous prime-boost immunization regimen with various types of anti-tumor immunity Human MART1 antigen presentation to mouse muscle cells injected with MART1 plasmid induces an antigen-specific immune response and MART1 antigen-specific memory T cells arise (priming). After MART1 plasmid injection, oncolytic adenovirus expressing MART1, mGM-CSF, and shmTGF-β2 were intratumorally injected (boosting). While the increased MART1 protein expression on tumor cells by adenovirus induces faster and stronger MART1-specific CD8^+^ and CD4^+^ T cell immune response, the adenovirus-mediated expression of mGM-CSF and suppression of TGF-β2 expression induce antigen-nonspecific activation of various immune cells. In this process, IFN-γ produced by active immune cells such as CD8^+^ T cells with the help of both of mature DCs and relieved regulatory T cells can trigger an anti-tumorigenic cytotoxicity. Furthermore, the oncolytic property of adenovirus induces viral replication in tumor cells and effective tumor cell lysis. Consequently, the virus can be released and re-infected surrounding tumor cells, and immune activation and tumor cell lysis are then repetitively induced by the virus.

In summary, Figure 8 provides a schematic diagram of prime-boost immunization by both the DNA vaccine encoding human MART1, and the oncolytic adenovirus expressing MART1, GM-CSF and shRNA of TGF-β2. Heterologous immunization induces a stronger anti-tumor effect through enhanced immune activation and tumor cell lysis.

## DISCUSSION

B16BL6 mouse melanoma cells express high levels of melanA; however, when these cells are injected into mice to induce tumor formation, the host immune tolerance to self-antigen (melanA) only permits a weak immune response to B16BL6 tumors [52]. To overcome this issue and induce an anti-B16BL6 tumor immune response, human MART1 plasmid was administered to mice as a DNA vaccine.

In this study, we evaluated the effectiveness of a prime-boost strategy in generating an antigen-specific protective response against the murine MART1 counterpart, which is endogenously expressed by murine melanoma B16BL6 cells, using plasmid DNA to express human MART1 antigen. The cross-species protective response may result from an epitope that is derived from the nearly 70% shared amino acid sequence or the 30% non-homologous amino acid sequence [49, 53]. The human MART1 sequence may contain peptides that efficiently bind to the C57BL/6 MHC class I molecules (H-2b) in muscle cells, and stimulate an effective response against murine MelanA expressed by B16BL6 cells [49]. To induce a stronger and longer-lived mouse melanoma antigen-specific immune response, the human MART1 plasmid and the recombinant adenovirus vector expressing MART1 were administered to tumor-bearing mice. Moreover, non-specific immune stimulation was induced by expression of GM-CSF, and inhibition of tumor cell survival, growth, and immune evasion was induced by siRNA of TGF-β. In the present study, shRNAs were used to suppress TGF-β1 and TGF-β2 expression, as these two isoforms are expressed in the majority of malignant melanomas [54, 55]. Although suppression of TGF-β expression was expected to strongly inhibit tumor growth and survival, the reduction of signaling molecules related to cell growth, survival, and metastasis was modest after downregulation of TGF-β1; however, the silencing of TGF-β2 resulted in a much more pronounced reduction in expression of these downstream signaling molecules.

The most effective way of anti-tumor immune activation was heterologous immunization with MART1-GMCSF-shTGFβ2 combination. Consistent with its ability to increase or decrease tumor-infiltrating immune cells (Figure 7C, 7D), or increase IFN-γ secretion (Figure 7E) or increase significantly active dendritic cells and decrease regulatory T cells (Figure 7G, 7H), MART1 and virus in Ad^MGshT^ treatment induced the greatest anti-tumor effect in immunecompetent mouse-model system suitable for our oncolytic adenovirus. Intriguingly, increases of splenic immune cells such as CD4^+^, CD8^+^ T and NKT cells or decrease of splenic CD4^+^CD25^+^FoxP3^+^ regulatory T cells after MART1 plasmid + Ad^MGshT^ were not significant compared to those of tumor-infiltrating immune cells, which provides the possibility that intratumoral injection can induce more rapid and potent immune activation in tumor site than in spleen.

One explanation as to why complete regression was not observed, even in the tumor tissue after treatment of MART1 plasmid + Ad^MGshT^, may be that the innate immune response to oncolytic adenovirus inhibits the spread and persistence of the virus within tumors via tumor-infiltrating immune cells, such as M2 macrophages [56, 57]. Another possibility is that the increased release of IFN-γ from tumor-infiltrating immune cells (Figure 7E) might have partially originated from the increase of antiviral effect, in addition to either T cell or NK cell activation, which eventually decreases the spreading of oncolytic adenovirus within tumors [57] and strengthens the gradual virus degradation (Figure 7F).

Taken together, these data imply that the combination of tumor antigen-specific induction via MART1, the non-specific immune stimulation via GM-CSF, the shTGF-β2-mediated anti-tumor effects, and the oncolytic function of adenovirus was more potent than the anti-tumor effects of individual treatment (MART1 or GM-CSF/shRNA of TGF-β2). Additionally, expected adverse effects such as autoimmune disease induced by excessive expression of multi-target genes can be strongly controlled by virus-oriented immune activation, resulting in multi genes-loaded oncolytic adenoviral degradation. Therefore, such combinations could be of use as better therapeutic strategies for controlling malignant melanoma.

## MATERIALS AND METHODS

### Cell culture

B16BL6 mouse melanoma cells were cultured in MEM with 10% fetal bovine serum FBS and MEM vitamin solution (HyClone, Logan, UT, USA). NIH-3T3 mouse embryo fibroblasts, B16F10 mouse melanoma cells, Lewis lung carcinoma (LLC) cells, and A375 human melanoma cells were cultured in DMEM with 10% FBS. Cells were maintained in a 37°C humidified atmosphere containing 5% CO_2_. Human melanoma SK-MEL-2, SK-MEL-3, and SK-MEL28 cells and human embryonic kidney 293A cells were cultured in RPMI-1640 with 10% FBS and maintained in a 37°C humidified atmosphere containing 5% CO_2_.

### Reagents

Antibodies to phosphoAkt, phosphoSrc, phosphoSTAT3, and β-catenin were purchased from Cell Signaling Technology (Beverly, MA, USA). Antibodies to MART1, MelanA, CD8, and actin were purchased from Santa Cruz Biotechnology (Santa Cruz, CA, USA). Antibodies to CD4, CD11b, CD25, NK1.1, IFN-γ and Ad5 were purchased from Novus Biologicals (Littleton, CO, USA). Antibody to CD11b+c was purchased from Thermo Fisher Scientific (Waltham, MA, USA). Antibodies to CD3-FITC, CD4-PE/Cy7, CD25-APC, and CD11c were purchased from Abcam (Cambridge, UK). Antibodies to CD122-PE/Cy7, CD8a-APC, FoxP3-PE, and NK1.1-APC were purchased from Biolegend (San Diego, CA, USA). All other chemicals were purchased from Sigma-Aldrich (St Louis, MO, USA).

### Construction of a stable cell line expressing CAR and E1B55KDa

After B16BL6 cells were transfected with a pIRES-CAR/E1B55 plasmid expressing both the adenovirus receptor CAR and the adenovirus protein E1B55 [47], cells were cultured in MEM with 10% FBS, MEM vitamin solution, and 0.5 mg/ml G418 (Calbiochem, La Jolla, CA, USA) as selection medium, and the media was changed every 2–3 days after transfection. The positive cell clones that expressed both CAR and E1B55KDa protein were selected and named B16BL6-CAR/E1B55. B16BL6-CAR/E1B55 cells were cultured in MEM with 10% FBS, MEM vitamin solution, and 0.5 mg/ml of G418, and cells were maintained in a 37°C humidified atmosphere containing 5% CO_2_.

### E1B-55 K polyclonal antibody production

A 25-mer synthetic peptide, MERRNPSERG VPAGFSGHASVESGC, from N-terminal of E1B-55K was selected among 5 candidates and conjugated to BSA as an immunogenic carrier protein and used to immunize two New Zealand White rabbits. The overall processes including antibody production and purification were performed by Young In Frontier (Seoul, Korea).

### Construction of replication-deficient GFP adenoviral vectors

The enhanced green fluorescent protein (EGFP) gene, which originated from pEGFP-N1 (Clontech Laboratories, Mountain View, CA, USA), was cloned into the pCA14 vector via digestion with XhoI/XbaI. After linearization by XmnI digestion, the construct was co-transformed into *Escherichia coli* BJ5183 together with the Bsp119I-digested adenoviral vector (dl324-BstBI, adenovirus vector with deleted E1 and E3 region) for homologous recombination. To verify the homologous recombination, plasmid DNA purified from overnight *E. coli* culture was digested with HindIII, and the digestion pattern was analyzed. The homologous recombinant adenoviral plasmid DNA was digested with PacI and transfected into 293A cells to generate replication-deficient adenovirus.

### Construction of mouse TGF-β1 and TGF-β2 shRNA

To construct mouse TGF-β1 shRNA, we screened eleven candidate sequences. Target selection was performed using an algorithm developed by Genolution Pharmaceuticals Inc. (Seoul, South Korea). The selected mouse TGF-β1 target sequence was 5’- CCCTCTACAACCAACACAACCCGGG-3’, and the loop sequence was 5’-TCTC-3’. To express mouse TGF-β1 shRNA in adenovirus, the top strand sequence (5’- GATC GCCTCTACAACCAACACAACCCGGGTCTCCCCGGGTTGTGTTGGTTGTAGAGGGTTTT-3’) and the bottom strand sequence (5’-AGCTAAAA CCCTCTACAACCAACACAACCCGGGGAGACCCGGGTTGTGTTGGTTGTAGAGGG-3’) were annealed and subcloned into the pSP72ΔE3-U6 E3 shuttle vector, which was digested with BamHI and HindIII. The resulting adenoviral shuttle vector, pSP72ΔE3-U6-shTGF-β1, was linearized by XmnI digestion. The adenoviral vector dl324-IX was linearized by SpeI digestion, and the two linearized vectors were co-transformed into E. coli BJ5183 cells to undergo homologous recombination. The recombined adenoviral plasmids, dl324-IX-ΔE3-U6-NC and dl324-IX-ΔE3-U6-shTGF-β1, were then digested with PacI and transfected into HEK-293 cells to generate the replication-incompetent adenovirus. For the construction of mouse TGF-β2 shRNA, target sequence (5’- GGATTGAACTGTATCAGATCCTTAA-3’) was selected from five candidate sequences, and the loop sequence was 5’-TCTC-3’. To express mouse TGF-β2 shRNA in adenovirus, the top strand sequence (5’-GATCCGGATTGAACTGTATCAGATCCTTAATCTCTTAAGGATCTGATACAGTTCAATCCTTTTA-3’) and the bottom strand sequence (5’-AGC TTAAAAGGATTGAACTGTATCAGATCCTTAAGAGATTAAGGATCTGATACAGTTCAATCCT-3’) were annealed and subcloned into the pSP72ΔE3-H1 vector. Then, the subsequent steps were performed as described for the production of mouse TGF-β1 shRNA-expressing adenovirus. The infectious titer of the adenovirus was determined by a limiting dilution assay in 293A cells.

### Construction of oncolytic adenoviral vectors

For the construction of oncolytic adenovirus expressing shRNA of mTGF-β1 or mTGF-β2, pSP72ΔE3-U6-shmTGFβ1 or pSP72ΔE3-H1-shmTGFβ2 was linearized by XmnI digestion and co-transformed into *E. coli* BJ5183 together with the SpeI-digested adenoviral vector (dl324-BstBI) for homologous recombination. The E1 shuttle vector was designed by subcloning HindIII-blunt-BglII-digested pBSK[3484] [58] into SspI-BglII-digested pCA14 for mTGF-β1 to become pCA14-3484-CMV-ΔE1B after replacement of the mouse survivin promoter with CMV promote by digestion with KpnI –XhoI and removal of the E1B55 promoter-E1B55-SV40 construct by an EcoRI-SalI blunt-ligation step. On the other hand, HindIII/EcoRI-digested pBSK[3484] was subcloned into HindIII/EcoRI-digested pVAX1 for the subcloning of mouse shRNA of TGF-β2. The E1R gene of adenovirus from StuI-blunt-EcoRI-digested pCA14 was inserted into ApaI-blunt-EcoRI-digested pVAX1-3484-ΔE1B vector. The final E1 shuttle vector for oncolytic adenovirus was constructed after the mouse survivin promoter in pBSK[3484] was replaced with the CMV promoter from pVAX1-3484-CMV-ΔE1B-E1R through digestion with KpnI and XhoI. The E1 shuttle vector was linearized by PmeI digestion, and co-transformed into *E. coli* BJ5183 together with the BstBI-digested dl324-BstBI-ΔE3-U6-shmTGFβ1 or BstBI-digested dl324-BstBI-ΔE3-H1-shmTGFβ2 for homologous recombination. For the construction of oncolytic adenovirus expressing shRNA of mTGF-β1 and mTGF-β2 in the same construct, the U6-shmTGFβ1-SV40 construct from pSP72ΔE3-U6-shmTGFβ1 was digested with SphI-blunt-KpnI and then subcloned into HindIII-blunt-KpnI-digested pSP72ΔE3-H1-shmTGFβ2 plasmid to yield pSP72ΔE3-H1-shmTGFβ2-U6-shmTGFβ1. Then, dl324-BstBI-H1-shmTGFβ2-U6-shmTGFβ1 was produced after the first homologous recombination followed by a second homologous recombination that resulted in generation of dl324-3484-ΔE3-H1-shmTGF-β2-U6-shmTGF-β1. For the expression of MART1, pVAX1-MART1, which was kindly provided by Dr. Butterfield (University of Pittsburgh, PA, USA), was used. The pMG-mGM-CSF, which was kindly provided by Dr. O'Sullivan (University College Cork, Ireland), was used for the subcloning of mGM-CSF. Each gene was subcloned into pIRES vector using NheI/MluI and XbaI/NotI, respectively. Then [CMVp-MART1-IRES], [CMVp-IRES-mGMCSF], and [CMVp-MART1-IRES-mGMCSF] constructs were subcloned into BglII/SalI-digested pVAX1-3484-CMV-ΔE1B vector, and these plasmids were named pVAX1-3484-CMV-ΔE1B-MART1, pVAX1-3484-CMV-ΔE1B-mGMCSF, and pVAX1-3484-CMV-ΔE1B-MART1-IRES-mGMCSF, respectively. These E1 shuttle vectors were linearized by PmeI digestion and then co-transformed into *E.coli* BJ5183 together with the Bsp119I-digested dl324-BstBI or dl324-BstBI-ΔE3-U6-shmTGFβ2 for homologous recombination, respectively.

### Flow cytometric analysis

After B16BL6-CAR/E1B55 cells were infected with recombinant adenovirus for two days, infected cells were trypsinized and washed twice with ice-cold PBS. Then cells were incubated with anti-MART1 antibody for 1 h at 4°C. After washing twice with ice-cold PBS, cells were incubated with Allophycocyanin (APC)-conjugated anti-mouse IgG (BD Biosciences, Lincoln Park, NJ, USA) antibody in the dark for 45 min at 4°C. Cells were then washed twice with ice-cold PBS. As a negative control, mouse IgG fluorescence control (BD Biosciences) antibody was used. Finally, cells were suspended again in PBS and analyzed using a flow cytometer. For the detection of various immune cells after combination treatment of MART1 plasmid with Ad^MGshT^, the following antibodies were used for staining: CD3-FITC, CD4-PE/Cy7, CD25-APC (Abcam, UK), and CD122-PE/Cy7, CD8a-APC, Foxp3-PE, NK1.1-APC (Biolegend, USA). After adding the appropriate antibody, the cells were incubated in the dark at 4°C for 30 minutes and washed 3 times by centrifugation using ice cold-PBS buffer, and then analyzed on a flow cytometer (Beckman Coulter, Inc., CA, USA). Data were analyzed by using FACSuit Software (BD, CA, USA).

### CPE assay

To evaluate the CPE of several tumor-selective replication-competent adenoviruses, cells were first plated at approximately 80% confluence into the wells of a 48-well tissue culture plate. The cells were then infected with replication-competent adenovirus at various MOIs. After 24 h, cells were monitored daily by microscopy. When cells exhibited lysis at the lowest MOIs, remaining cells on the plate were fixed with 4% paraformaldehyde and stained with 0.05% crystal violet for analysis.

### Non-radioactive cytotoxicity assay (LDH assay)

The activity of splenocytes was assessed via an LDH assay using the non-radioactive cytotoxicity assay kit (Promega, Madison, WI, USA). Cancer cells were incubated for 12 h in 48-well plates at 37°C under a humidified atmosphere of 5% CO_2_ and then co-cultured with splenocytes isolated from C57BL/6 mice for 4 h. For the LDH-positive control, cells were incubated for 45 min with 45 μl of lysis solution (10X) to lyse the cells. After 45 min, the plates were centrifuged at 250 × *g* for 4 min, and then 50 μl of supernatant from each well was transferred to a fresh 96-well flat-bottom plate. A total of 50 μl of reconstituted Substrate Mix was added to each well of the plate, and the plate was then incubated for 30 min at room temperature in the dark. After 30 min, 50 μl of Stop Solution was added to each well, and the absorbance was recorded at 490 nm within 1 h using an ELISA plate reader (Molecular Devices Corporation, Sunnyvale, CA, USA). The percent cytotoxicity of adenovirus was calculated by the following formula:

% Cytotoxicity = ([experimental value - effector control value - negative control value] / [positive control value - negative control value])*100.

### Real-time PCR

After B16BL6-CAR/E1B55 cells were infected with the recombinant adenovirus for 2 days, cells were lysed with the Trizol reagent (Life Technologies, Carlsbad, CA, USA), and the total RNA was isolated using chloroform. The RNA concentration was determined using the Nanodrop 2000 (Thermo Fisher Scientific). The real-time PCR reaction was performed using the Power SYBR Green RNA-to-CT 1-Step Kit (Life Technologies). The reaction mixture contained the reverse transcriptase enzyme mix, reverse transcription PCR mix, forward primer, reverse primer, RNA template, and nuclease-free water. Mouse TGF-β1 cDNA was amplified using the forward primer 5′- TTGCTTCAGCTCCACAGAGA -3′ and the reverse primer 5′- TGGTTGTAGAGGGCAAGGAC -3′. Mouse TGF-β2 cDNA was amplified using the forward primer 5′-GTGAATGGCTCTCCTTCGAC-3′ and the reverse primer 5′-CCTCGAGCTCTTCGCTTTTA-3′. Mouse TGF-β3 cDNA was amplified using the forward primer 5′- CTATCAGGTCCTGGCACTTT-3′ and the reverse primer 5′- GGCAGATTCTTGCCACCTAT -3′. Mouse β-actin was amplified using the forward primer 5′-GGCTGTATTCCCCTCCATCG-3′ and the reverse primer 5′-CCAGTTGGTAACAATGCCATGT-3′.

### ELISA

Cells were plated in the wells of six-well plates at 2 × 10^5^ cells/well and then infected with adenovirus (C, G, shT, GshT, or MGshT virus) at an MOI of 50. Infected cells were incubated for one more day after changing to serum-free medium after 24 h infection and the ELISA was performed after harvesting the medium. The levels of GM-CSF or TGF-β2 expression were determined by an ELISA according to the manufacturer's instructions (R&D Systems, Minneapolis, MN, USA).

### Murine spleen cell preparation

One week after the final injection of [pVAX1 (control plasmid) or pVAX1-MART1 plasmid] or [pVAX1-MART1 plasmid with Ad or pVAX1-MART1 plasmid with Ad^MGshT^], C57BL/6 mice were sacrificed, and their spleens were extracted. After extraction, spleens and 1 ml PBS were placed directly into a cell strainer in a petri dish. The spleens were mashed using the black rubber portion of the syringe, and splenocytes were released into the petri dish. The homogenized cell suspension was then washed two times with PBS. Splenocytes were resuspended in 4 ml PBS per spleen, and then ammonium chloride-based lysing reagent (BD Biosciences) was added. Cells were then incubated for 15 min in the dark at room temperature. Cells were washed two times with PBS and then resuspended in RPMI-1640.

### Animal studies

The animal protocol (2014-0335) used in this study was reviewed and approved by the Institutional Animal Care and Use Committee in Yonsei University Health System.

Tumors were implanted subcutaneously in the abdomens of C57BL/6 mice by injecting B16BL6-CAR/E1B55 murine melanoma cells (7 × 10^5^) in 100 μl of Hank's balanced salt solution (HBSS; Gibco BRL). When tumors reached a range of 50-60 mm^3^, animals were randomized into four groups (PBS, Ad, Ad^G^, and Ad^GshT^) of 5 animals each. Adenovirus or PBS were administered intratumorally (virus; 1 × 10^9^ plaque-forming unit (PFU) per tumor in 50 μl of PBS) on days 1, 3, and 5. Tumors were implanted subcutaneously in the abdomens of C57BL/6 mice by injecting B16BL6-CAR/E1B55 murine melanoma cells (7 × 10^5^) in 100 μl of HBSS (Gibco BRL). After 4 days, C57BL/6 mice were injected intramuscularly in the rear quadriceps with 50 μg of pVAX1-MART1 encoding MART1 (M) in a total volume of 50 μl saline using a 29-gauge needle. When tumors reached a range of 50-60 mm^3^, animals were randomized into 5 groups (M+PBS, M+Ad, M+Ad^M^, M+Ad^GshT^, and M+Ad^MGshT^) of 5 animals each, and treatment was initiated. The first day of treatment was designated as day 1. Adenovirus or PBS was administered intratumorally (1 × 10^9^ PFU per tumor in 50 μl of PBS) on days 1, 3, and 5. Regression of tumor growth was assessed by taking measurements of the length (L) and width (W) of the tumor. Tumor volume was calculated using the following formula: volume = 0.52 x L x W^2^.

### Immunohistochemistry (IHC)

IHC studies were performed on paraffin-embedded tumor tissues using anti-CD4, anti-CD8, anti-NK1.1, anti-CD11b+c, anti-IFN-γ and anti-Ad5 antibodies to determine the expression of these proteins in the tumor tissues. The tumor tissue slides were deparaffinized by incubating in xylene for 10 min and rehydrated serially in alcohol (100%, 90%, and 70%). Endogenous peroxidase was blocked by incubation with 3% H_2_O_2_ for 15 min at room temperature, and antigen retrieval was achieved by incubating the slides in citrate buffer for 10 min in a steamer. For permeabilization, the slides were incubated in PBS containing 0.5% Triton X-100 for 30 min and then washed three times with PBS. To reduce non-specific background staining due to endogenous peroxidases, the slides were incubated with a hydrogen peroxide block (Thermo Scientific) for 10 min. After washing, an ultra V block (Thermo Scientific) was applied to the slides for 5 min at room temperature to further block non-specific background staining. The slides were incubated with an anti-CD4 antibody (1:200 dilution), an anti-CD8 antibody (1:500), anti-NK1.1 antibody (1:500), anti-CD11b+c antibody (1:500), anti-IFN-γ antibody (1:200) and anti-Ad5 antibody (1:800 dilution) for 12 h at 4°C and then further with a horseradish peroxidase polymer (Thermo Scientific) for 15 min at room temperature. To detect protein expression, the tissue sections were stained with diaminobenzidine tetrahydrochloride and minimally counterstained with hematoxylin (for visualization of antigen–antibody complexes). Sections were mounted under a coverslip using an aqueous mounting solution (Shandon Synthetic Mountant (Thermo Scientific) and xylene at a 1:1 ratio.

### Confocal immunofluorescence staining

Immunofluorescence double staining was performed on paraffin-embedded tumor tissues using anti-CD4 (Novus Biologicals, USA), anti-CD25 (Novus Biologicals, USA), anti-CD11c (Abcam, UK) and anti-CD11b (Novus Biologicals, USA) antibodies to determine the regulatory T cells and tolerogenic DC cells in the tumor tissues. The tumor tissue sections were deparaffinized by incubating xylene for 10 min and rehydrated serially in alcohol (100%, 90%, and 70%), and antigen retrieval was achieved by incubating the slides in citrate buffer for 10 min in a steamer. For permeabilization, the sections were incubated in PBS containing 0.5% Triton X-100 for 30 min and then washed three times with PBS. To reduce non-specific background staining the sections were incubated with 10% FBS for 1 hour in room temperature, and then incubated in 1^st^ primary antibody (CD4, CD11c) at appropriate dilution (1:2000) in PBS for 20 min at room temperature. After washing with PBS containing 0.1% Tween-20 for 3 times, the sections were incubated in flamma 488-conjugated secondary antibody (1:1000) (BioActs, Korea) in PBS for 10 minutes at room temperature. After 2^nd^ blocking process, the sections were incubated in 2^nd^ primary antibody (CD25, CD11b) at appropriate dilution (1:2000) in PBS for 1 hour at room temperature. After washing with PBS containing 0.1% Tween-20 for 3 times, the sections were incubated in flamma 552-conjugated secondary antibody (1:1000) (BioActs, Korea) in PBS for 10 minutes at room temperature. Finally, the sections were counterstained and coverslipped with DAPI-Fluoromount-G (Aviva, San Diego, USA) for 20 minutes at room temperature to stain nuclei. Images were acquired using Confocal laser scanning microscope (Carl Zeiss, Jena, Germany).

### Statistical analysis

The data were expressed as mean ± standard error (SE). Statistical comparison was made using SigmaPlot 8.0 (Systat Software Inc., San Jose, CA, USA). *P*-values less than 0.05 were considered statistically significant (*, *P*<0.05; **, *P*<0.01; ***, *P*<0.001).

## Abbreviations

MART1: melanoma antigen recognized by T cells 1
GM-CSF: granulocyte-macrophage colony-stimulating factor
TGF-β: transforming growth factor-β
CTLA4: cytotoxic T lymphocyte-associated protein 4
FACS: fluorescence-activated cell sorter
FBS: fetal bovine serum
NK: natural killer cell
DC: dendritic cell
MDSC: myeloid derived suppressor cells
PBS: phosphate-buffered saline
shRNA: short hairpin RNA
LDH: lactate dehydrogenase
CTL: cytotoxic T lymphocyte
APC: allophycocyanin
TIDC: tumor-infiltrating dendritic cell
TIL: tumor-infiltrating lymphocytes
